# Genetic Dissection of a Precocious Phenotype in Male Tiger Pufferfish (*Takifugu rubripes*) using Genotyping by Random Amplicon Sequencing, Direct (GRAS-Di)

**DOI:** 10.1007/s10126-020-10013-4

**Published:** 2021-02-18

**Authors:** Sota Yoshikawa, Masaomi Hamasaki, Kazushi Kadomura, Toshiyuki Yamada, Hisashi Chuda, Kiyoshi Kikuchi, Sho Hosoya

**Affiliations:** 1 Nagasaki Prefectural Institute of Fisheries, Nagasaki, Japan; 2 Fisheries Laboratory, Graduate School of Agricultural and Life Sciences, University of Tokyo, Shizuoka, Japan; 3 Aquaculture Research Institute, Kindai University, Wakayama, Japan

**Keywords:** GRAS-Di, Family structure analysis, Linkage map, Quantitative trait locus, Tiger pufferfish, Precociousness

## Abstract

The novel non-targeted PCR-based genotyping system, namely Genotyping by Random Amplicon Sequencing, Direct (GRAS-Di), is characterized by the simplicity in library construction and robustness against DNA degradation and is expected to facilitate advancements in genetics, in both basic and applied sciences. In this study, we tested the utility of GRAS-Di for genetic analysis in a cultured population of the tiger pufferfish *Takifugu rubripes*. The genetic analyses included family structure analysis, genetic map construction, and quantitative trait locus (QTL) analysis for the male precocious phenotype using a population consisting of four full-sib families derived from a genetically precocious line. An average of 4.7 million raw reads were obtained from 198 fish. Trimmed reads were mapped onto a Fugu reference genome for genotyping, and 21,938 putative single-nucleotide polymorphisms (SNPs) were obtained. These 22 K SNPs accurately resolved the sibship and parent–offspring pairs. A fine-scale linkage map (total size: 1,949 cM; average interval: 1.75 cM) was constructed from 1,423 effective SNPs, for which the allele inheritance patterns were known. QTL analysis detected a significant locus for testes weight on Chr_14 and three suggestive loci on Chr_1, Chr_8, and Chr_19. The significant QTL was shared by body length and body weight. The effect of each QTL was small (phenotypic variation explained, PVE: 3.1–5.9%), suggesting that the precociousness seen in the cultured pufferfish is polygenic. Taken together, these results indicate that GRAS-Di is a practical genotyping tool for aquaculture species and applicable for molecular breeding programs, such as marker-assisted selection and genomic selection.

## Introduction

Recent advances in genomic tools have been proven to be very useful not only for ecological and evolutionary studies but also for the agricultural sciences. Notably, developments in various high-throughput cost-effective genotyping-by-sequencing (GBS) systems, such as RADseq (and its derivatives) (Baird et al. 2008; Peterson et al. 2012; Andrews et al. 2016), MIGseq (Suyama and Matsuki 2015), and Ampliseq (Sato et al. 2019), facilitate a wide range of genetic analyses, including population structure analysis (Hohenlohe et al. 2010; Cavender-Bares et al. 2015; Hirase et al. 2020), linkage map construction (Elshire et al. 2011; Hoshino et al. 2016; Escudero et al. 2018), and forward genetics (Wilson et al. 2014; Zhou et al. 2015; Ieda et al. 2018; Kim et al. 2019), even for non-model organisms. These technologies are also recognized as powerful tools for aquaculture studies, as they are utilized in the implementation of selective breeding programs and the management of genetic diversity of target populations (Houston et al. 2012; Palaiokostas et al. 2013; Hosoya et al. 2018).

As an addition to these GBS tools, a new technology named Genotyping by Random Amplicon Sequencing, Direct, was developed recently **(**Hosoya et al. 2019). GRAS-Di is a non-targeted PCR-based GBS system, consisting of two sequential PCRs and a final purification step, as in the other non-targeted PCR base GBS system, namely multiplexed Inter-Simple Sequence Repeat (ISSR) genotyping by sequencing (MIG-seq, Suyama and Matsuki 2015). As variations of GBS, both GRAS-Di and MIG-seq have the advantage of reduced representation sequencing methods, and the cost, sample size, and the number of SNPs are optimally balanced. The major advantage of these methods is that they permit the use of small amounts of fragmented DNA (< 100 ng), unlike RADseq, which requires a large amount of high-quality DNA (> 1 µg; Etter et al. 2011; Hohenlohe et al. 2011). The main differences between GRAS-Di and MIG-seq are the first PCR primer. In the case of MIG-seq, the first PCR primers for MIG-seq, including 12-bp SSR sequences with 2-bp anchor oligos at 3′ tail, are designed to amplify non-repetitive regions between SSRs. Therefore, only a limited number of SNPs can be genotyped because of the low frequency of SSRs over the genome (< 1000; Watanabe et al. 2018). On the other hand, the first PCR primers of GRAS-Di consist of 10-bp Illumina Nextera adaptor plus 3-bp random oligomers at the 3′-end, making it possible to amplify several thousands of loci even with such a simple library construction procedure, from various organisms. The availability for population structure analysis has been confirmed using mangrove fishes (Hosoya et al. 2019), alpine snow-bed herb (Ikeda et al. 2020), and Tsushima leopard cat (Ito et al. 2020). Because of its simplicity in library preparation and robustness against DNA degradation, it is expected that this technology will be applicable for a wide range of genetic studies in basic and applied sciences. However, the potential of GRAS-Di for genetic analysis is not yet fully investigated.

In this study, we tested the utility of GRAS-Di in aquaculture studies using the tiger pufferfish *Takifugu rubripes*. Known as Fugu, this fish has served as a model species for genomic studies since the first draft reference genome was published (Aparicio et al. 2002). A high-quality genomic reference (i.e., assembled at the chromosome level) is now available (Kai et al. 2011) and has been applied to various genetic studies (Kamiya et al. 2012; Hosoya et al. 2013; Ieda et al. 2018). The species is also known for the high market price and is recognized as one of the important aquaculture species in East Asia (Wang et al. 2016; Hamasaki et al. 2017). Although the fish itself is valuable, a particularly prized part of the fish in Japan is the testes. Testes size is a valuable economic trait, since mature testes, known as “shirako”, are regarded as a delicacy in Japan (Hamasaki et al. 2013). Recently, a family line which is genetically precocious, i.e., early onset of testicular development about 2 months prior to the other lines, was identified (Yoshikawa et al. 2020). However, the genetic architecture of the precocious phenotype has not yet been studied.

The aim of this study is three-fold: (1) to test the utility of SNPs obtained by GRAS-Di for a family structure (parentage) analysis using a cultured population of the tiger pufferfish, (2) to evaluate the applicability of these SNPs for genetic map construction, and (3) to investigate the feasibility of the linkage map to dissect the genetic architecture of the precocious traits by means of quantitative trait locus (QTL) analysis. For this purpose, we raised four full-sib families, grand-offspring of a precocious male and a non-precocious female, and applied GRAS-Di on them.

## Materials and Methods

## Experimental Crosses for Genetic Analysis

We produced four full-sib families that were descendants of a grandfather (GF) and a grandmother (GM). The GF individual was derived from one of the major domesticated family lines in Japan and identified as genetically precocious by progeny tests (Yoshikawa et al. 2020), while GM belonged to another major line not characterized as precocious. These grandparents were crossed in March 2012 at private hatchery in Nagasaki Prefecture, Japan, to obtain the first generation (*F*_1_) (Table 1). *F*_1_ individuals were raised at NPIF. Two males to be used as fathers (FA1 and FA2) and two females as mothers (MO1 and MO2) were chosen randomly and crossed in a diallelic manner in April 2015 at NPIF to produce four full-sib families of the second generation (*F*_2_) (from Fam1 to Fam4, Table 2). The caudal fin was clipped from GF and the four parental fish. Unfortunately, a tissue sample for GM was not available from the commercial hatchery. These tissue samples were stored in 99.5% ethyl alcohol at − 30 °C for DNA extraction.

**Table 1:** Relevant details of the parental fish used in this study

ID	Sex	Background	Mating information			
			Production	Date	Age (years)	BW^a^ (kg)
GF	Male	Genetically precocious sire	*F*_1_	30-Mar-2012	2	1.8
GM	Female	Derived from a major domesticated (non-precocious) family line			4	3.7
FA1	Male	Selected randomly from F_1_ individuals	*F*_2_	7-Apr-2015	3	3.3
FA2	Male	Same as above			3	2.5
MO1	Female	Same as above			3	2.4
MO2	Female	Same as above			3	2.8

^a^Body weight

**Table 2:** Relevant details regarding the identity of the parents, number of fish transferred to the communal tank (*n*), average standard length (SL), and body weight (BW) at tagging of each *F*_2_ family

Family ID	Sire	Dam	*n*	SL (cm)^a^	BW (g)^a^
Fam1	FA1	MO1	120	16.4 ± 1.1	132.6 ± 23.6
Fam2	FA2	MO1	120	16.3 ± 1.0	134.7 ± 20.9
Fam3	FA1	MO2	120	17.0 ± 0.8	145.2 ± 18.7
Fam4	FA2	MO2	120	16.0 ± 1.1	129.7 ± 23.0

^a^Values are mean ± SD

## Culture in Communal Tanks

All test families were obtained by in vitro fertilization and reared following the recommendations of Yoshikawa et al. (2020). Each full-sib family of the *F*_2_ generation was cultured separately until the mean standard length (SL) reached approximately 160 mm and mixed in a communal tank in September 2015. Before mixing, 120 individuals were randomly sampled from each full-sib family and each fish was fitted with a passive integrated transponder (PIT) tag (Bio Mark, ID, USA). Average SL and body weight (BW) at transfer to the communal tank are given in Table 2. In the communal tank, the fish were fed commercial pellets four to seven times a week until satiation. The holding tank was supplied with UV-sterilized ambient seawater. In December 2016, all the surviving fish were euthanized using an overdose of 2-phenoxyethanol (> 600 ppm) (Fujifilm Wako Pure Chemical, Osaka, Japan). Survival rates of each family were in the range of 78.3 to 87.5%. Each individual was visually sexed, and only males were used for the following analysis. SL and BW were measured, and the testes were excised and weighed. The gonadosomatic index [GSI = 100 × gonad weight (g) / total body weight (g)] was calculated for each male. The Spearman’s rank order correlations were used to assess the strength of association among the four traits. The caudal fin was clipped from each specimen and stored in 99.5% ethyl alcohol at − 30 °C for DNA extraction.

## Genotyping

Genomic DNA was extracted from the caudal fin using a DNeasy Blood and Tissue Kit (Qiagen, Hilden, Germany) according to the manufacturer’s instructions. Library preparation and sequencing for GRAS-Di were done as described in Hosoya et al. (2019) by Eurofins Genomics Inc. (Tokyo, Japan). In short, template genomic DNA obtained from each sample was amplified with the first PCR primers including Illumina Nextera adaptor sequences and three-base random oligomers, followed by the second PCR using indexing primers consisting of the Illumina multiplexing dual index and P7/P5 adapter sequence. The final PCR products were pooled to produce two independent libraries (*n* = 100 and 98, respectively) and purified using the MiniElute PCR Purification Kit (Qiagen). These libraries were sequenced (one library per lane) on a HiSeq 2500 platform (Illumina, CA, USA), with 100 bp paired-end reads. The sequence data have been registered in the DDBJ Sequence Read Archive database (Accession No. DDBJ: DRA010711, Online resource 1).

To call genotypes, trimmed reads were mapped on to a Fugu reference genome assembly (FUGU5/fr3) (Kai et al. 2011). Trimming was done using Trimmomatic‐0.36 (Bolger et al. 2014) by setting the parameters as follows: ILLUMINACLIP NexteraPE-PE.fa:2:30:10, SLIDINGWINDOW:30:20, AVGQUAL:20, and MINLEN:80. Trimmed reads were mapped onto the reference using BWA-mem of BWA v 0.7.17 (Li 2013) with default parameters. Subsequently, mapped bam files were combined using Samtools v 1.9 (Li et al. 2009) and SNP calling was done by joint analysis using Freebayes v 1.3.1-17 (Garrison and Marth 2012, available at https://arxiv.org/abs/1207.3907) with options of *--min-mapping-quality 10*, *--use-best-n-alleles 4*, and *--min-alternate-fraction 0.2*. We excluded insertion/deletion mutations and low-quality SNPs using vcftools v 1.17 (Danecek et al. 2011) with the following criteria, genotyped in < 80% of individuals, read quality < 20, minor allele frequency < 0.01, with more than two alleles, read depth < 5, and average read depth > 500.

## Genetic Analysis

First, the population structure was inferred from the putative SNPs using principal component analysis (PCA) implemented in PLINK2.0 (Chang et al. 2015). Next, effective SNPs for which the allele inheritance (either from GF or GM) was known were extracted and a genetic linkage map for QTL analysis constructed. In this analysis, since it was not possible to obtain a tissue sample for GM from the commercial hatchery, the pattern of allele inheritance at many loci is ambiguous. However, the allelic state of the loci where GF’s genotype was homozygous while those of the four parents (FA1, FA2, MO1, and MO2) were heterozygous could be determined even without GM’s genomic information since the allele inherited from GF was known. Such SNP loci were considered effective in this study. Since these SNPs were available for all families, we were able to construct a single genetic map, with genetic distances between loci averaged over the four families. SNPs were further excluded based on the following criteria: (1) SNPs for which chromosomal position was unknown, (2) SNPs called in less than 98% of the individuals, and (3) SNPs that deviated from the Hardy-Weinberg equilibrium (*p* < 10^−8^), except for those on chromosome 19. We note that Chr_19 is the sex chromosome of the tiger pufferfish (Kamiya et al. 2012), and thus, segregation of SNPs on this chromosome is expected to depart from Hardy-Weinberg equilibrium in this analysis because only males were used. Map distances between SNPs were calculated using Kosambi’s map function implemented in R/qtl (Broman et al. 2003). When the map distance between two adjacent SNPs was larger than 50 cM, the downstream SNP was excluded because genotypes of these SNPs were possibly miscalled.

Genome-wide QTL analysis was done using the *scanone* function of R/qtl package by interval mapping (1 cM step) with the expectation-maximization (EM) algorithm. Although the family-based culture period was short (5 months) compared with the subsequent communal culture (15 months), there was the possibility of unexpected environmental effects from the family-based culture. The tank effect was included as an additive covariate in the model. Phenotypic values of SL, TW, and GSI, which were not distributed normally, were transformed to the corresponding normal quantiles using the *nqrank* function. The genome-wide thresholds for suggestive (*p* < 0.68 = 1–0.95^22^) and significant (*p* < 0.05) QTLs were determined by means of 1,000 permutations. The suggestive threshold was adopted from Kirschner et al. (2012), considering that the tiger pufferfish has 22 pairs of chromosomes (Kai et al. 2011). The 95% credible interval (CI) of the QTL was determined by the *bayesint* function. Phenotypic variation explained (PVE) (%) by the QTL was estimated by means of a drop-one-term analysis following multiple QTL model fitting via a Haley-Knott regression. Phenotype and genotype data for QTL analysis was listed in Online resource 1.

## Candidate Gene Search

To identify candidate genes responsible for the phenotypes, we searched genes within the 95% CI of the significant QTL regions from the Fugu reference genome databases (Fugu5/fr3) archived in UCSC Table Browser (https://genome.ucsc.edu, accessed May 25, 2020) and retrieved a list of Ensembl Genes. The biological function of each gene was checked using the NCBI Entrez Gene database (https://www.ncbi.nlm.nih.gov/gene, accessed June 7, 2020), and genes relating to maturation and growth, such as steroidogenic enzymes, gonadotropin releasing hormone, gonadotropin, and genes with cell proliferation and bone formation functions.

## Results

### Phenotype Summary

Sample number, average SL, BW, testes weight (TW), and GSI of male progeny are summarized in Table 3. BW was normally distributed (Shapiro-Wilk test, *p* < 0.05), but SL, TW, and GSI were not. A significant correlation was observed between each pair of traits (Spearman’s *p* < 0.05) (Table 4).

**Table 3:** Number of samples (*n*), standard length (SL), body weight (BW), testes weight (TW), and gonadosomatic index (GSI) of the males in each *F*_2_ family used for genetic map construction and quantitative trait loci analysis

Family ID	*n*	SL (cm)^a^	BW (g)^a^	TW (g)^a^	GSI^a^
Fam1	45	33.8 ± 2.1	1128.4 ± 209.1	51.3 ± 43.2	4.2 ± 3.1
Fam2	50	33.2 ± 1.8	1071.4 ± 166.5	52.8 ± 32.3	4.7 ± 2.5
Fam3	42	34.3 ± 2.0	1189.0 ± 215.7	46.3 ± 33.5	3.7 ± 2.4
Fam4	56	32.8 ± 2.5	1056.3 ± 231.5	30.5 ± 27.4	2.6 ± 2.0

^a^Values are mean ± SD

**Table 4:** Pairwise correlation for standard length (SL), body weight (BW), testes weight (TW), and GSI. Spearman’s correlation coefficients (*ρ*) are shown in the lower half and *p* values in the upper half of the matrix

	SL	BW	TW	GSI
SL		< 0.0001	< 0.0001	< 0.0001
BW	0.917		< 0.0001	< 0.0001
TW	0.583	0.695		< 0.0001
GSI	0.478	0.582	0.986	

## Genotyping

In total, 24.2 million and 905.0 million reads were generated for the parental fish (GF, FA1, FA2, MO1, and MO2) and 193 *F*_2_ males by GRAS-Di, respectively. After trimming, 81.1% of the reads were retained on average. These trimmed reads were mapped onto the reference genome and applied for joint genotype calling. This yielded 633,818 variants. After filtering, 21,938 putative SNPs were retained.

## Family Structure Analysis

**Fig. 1:**
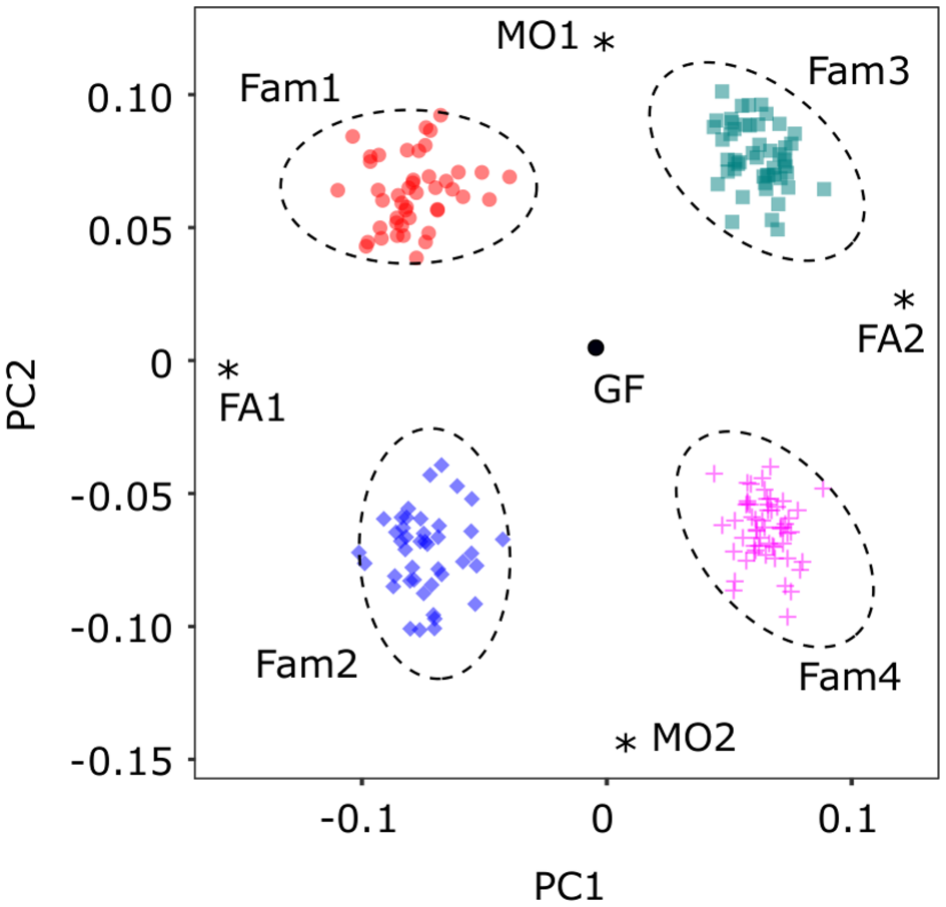
Principal component analysis (PCA) plot for the genetic population structure of the test family, with the following individuals being represented: the grandfather (GF:  ), *F*_1_ parents (FA1, FA2, MO1, and MO2: ⁎), and *F*_2_ siblings from Fam1 (  ), Fam2 (  ), Fam3 (  ), and Fam4 (  ). The dotted circle indicates each full-sib family tracked from PIT tag IDs

These 22 K SNPs could detect the family structure successfully (Fig. 1). Individuals from each *F*_2_ family clustered together and the four parents placed between the siblings, while GF plotted in the middle of the descendants. Parentage assignments based on the plot coincided perfectly with the family records based on PIT tag IDs.

## Linkage Map Construction

**Fig. 2:**
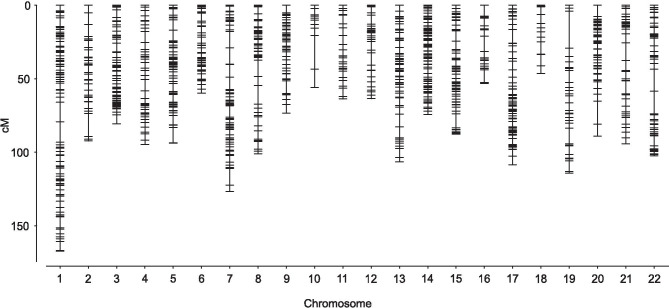
The genetic linkage map constructed from the test cross of the tiger pufferfish (1,423 SNPs). Chromosomes are on the *x*-axis and genetic distance is shown on the *y*-axis

A total of 2,491 effective SNPs were detected where the genotype of GF was homozygous and that of each of the parents (FA1, FA2, MO1, and MO2) was heterozygous. Subsequent filtration steps excluded 1,062 SNPs, with 434 SNPs excluded because the genomic positions of these SNPs could not be determined, 530 because of low genotyping rate (< 98%), and 98 deviated from the Hardy-Weinberg equilibrium (*p* < 10^−8^). In total, 1,429 effective SNPs were obtained. Among these SNPs, six were excluded based on the map distance criterion (> 50 cM between adjacent SNPs). Thus, the linkage map was constructed from the remaining 1423 SNPs (Fig. 2 and Online resource 2). The map spanned 1,949.2 cM, ranging from 46.3 (Chr_18) to 167.4 cM (Chr_1), and the average intervals between SNPs were 1.75 cM (with a maximum of 3.99 cM on Chr_9). The order of SNPs on the linkage map was in accordance with that on the reference genome.

## QTL Analysis

**Fig. 3:**
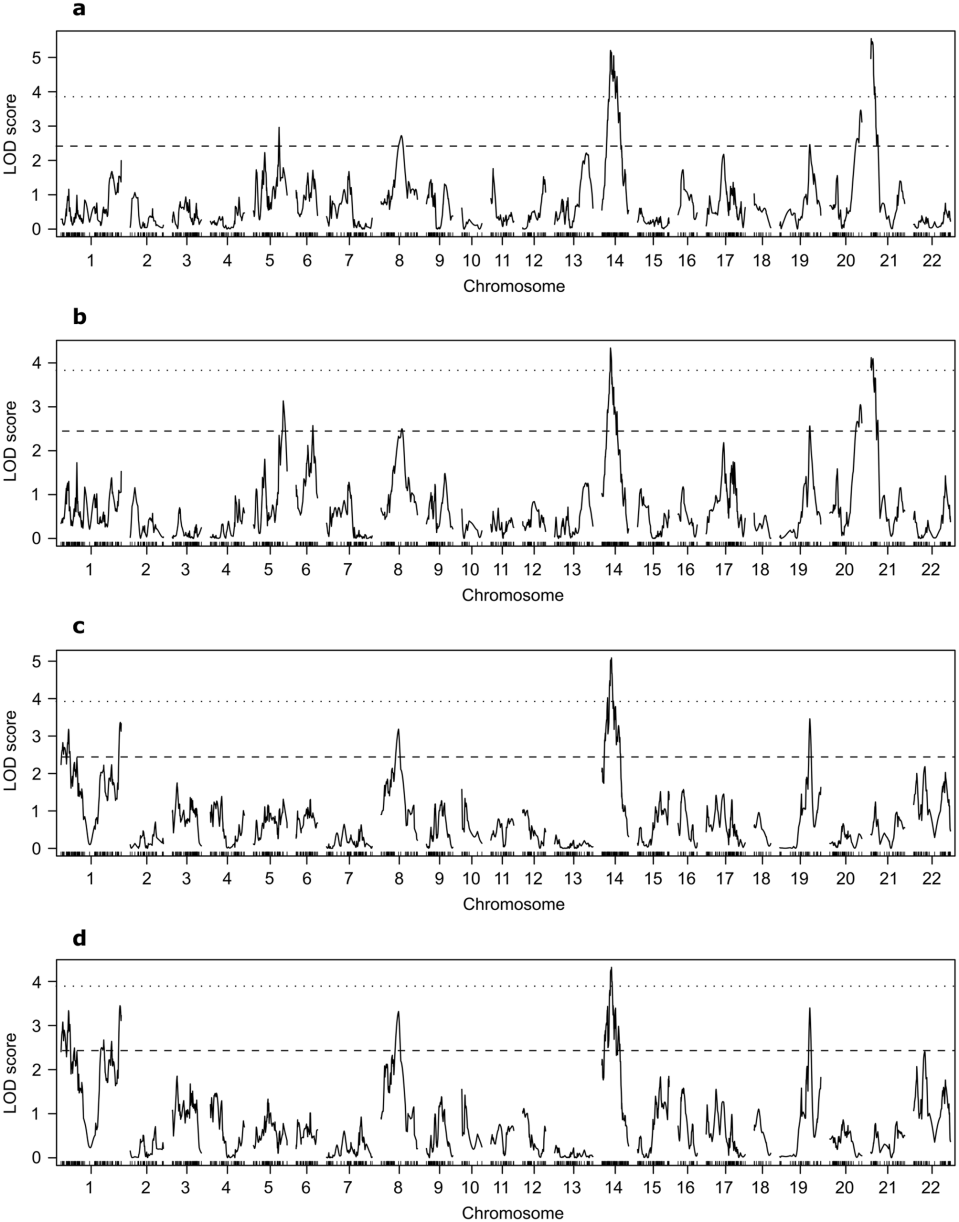
Results of quantitative trait loci analysis of the tiger pufferfish for **a** standard length, **b** body weight, **c** testes weight, and **d** gonadosomatic index. Log of odds (LOD) scores are plotted over 22 chromosomes. Dotted and dashed horizontal lines indicate genome-wide significant (*p* < 0.05) and suggestive (*p* < 0.68) levels of LOD scores, respectively

The genome-wide significant levels (*p* < 0.05) of log of odds (LOD) score, determined by 1,000 times permutation test, were 3.82, 3.98, 4.02, and 3.95 for SL, BW, TW, and GSI, respectively. Significant QTLs were detected for each trait (Fig. 3 and Table 5). The direction of additive and dominant effects of significant QTLs were consistent and alleles inherited from GM showed negative effects. Interestingly, body and testes size shared a QTL on Chr_14, where the 95% CI of the QTL overlapped each other. The two body size traits (SL and BW) shared six QTLs, but the suggestive QTL (*p* < 0.68) on Chr_6 was only detected for BW. The effect of each QTL (i.e., PVE) was small even for the significant QLTs, as it ranged 5.4 to 10.5%. TW and GSI shared all QTLs. LOD scores and PVE of the significant QTL were 5.1 and 5.9% for TW and 4.3 and 4.8% for GSI, respectively.

**Table 5:** Summary of quantitative trait loci (QTL) analysis for standard length (SL), body weight (BW), testes weight (TW), and gonadosomatic index (GSI)

Trait	Chromosome	Position (cM)	SNP_ID^a^	LOD^b^	95% CI^c^		PVE ^d^ (%)	Add^e^	Dom^f^
					Genetic map (cM)	Physical map (Mb)			
SL	Chr_5	71.5	Chr_5:10,219,766	2.96	2.3–93.5	0.22–11.83	5.02	0.55	0.77
	Chr_8	56.0	Chr_8:10,920,029	2.72	31.4–97.9	8.95–13.61	1.03	-0.19	0.38
	**Chr_14**	**23.9**	**Chr_14:4,678,973**	**5.20**	**21.0–43.3**	**4.14–7.89**	**6.42**	**-0.81**	**-0.15**
	Chr_19	83.6	Chr_19:10,727,069	2.46	3.9–113.1	0.32–13.67	3.45	-1.08	0.33
	Chr_20	86.0	Chr_20:13,312,894	3.47	65.2–89.1	10.94–13.31	3.13	0.69	0.10
	**Chr_21**	**1.0**	**Chr_21:96,184**	**5.55**	**0.0–7.6**	**0.05–0.75**	**10.50**	**-0.84**	**-0.78**
BW	Chr_5	83.1	Chr_5:11,221,461	3.13	29.0–93.8	3.79–12.00	8.37	84.05	35.2
	Chr_6	46.7	Chr_6:7,686,560	2.57	0.0–57.1	0.26–8.50	4.94	27.02	-92.33
	Chr_8	58.0	Chr_8:10,920,029	2.50	27.6–91.0	8.48–12.97	0.69	-18.10	30.04
	**Chr_14**	**23.9**	**Chr_14:4,678,973**	**4.34**	**15.5–42.1**	**2.44–7.88**	**5.37**	**-72.50**	**-13.62**
	Chr_19	83.6	Chr_19:10,727,069	2.56	58.0–113.1	6.20–13.67	3.63	-87.10	6.98
	Chr_20	85.0	Chr_20:12,076,849	3.05	65.2–89.1	10.94–13.31	3.21	67.42	11.99
	**Chr_21**	**1.0**	**Chr_21:96,184**	**4.12**	**0.0–13.7**	**0.05–3.70**	**7.00**	**-74.96**	**-45.83**
TW	Chr_1	165.0	Chr_1:22,807,065	3.37	0.0–167.4	0.17–22.90	3.14	-8.27	7.36
	Chr_8	48.6	Chr_8:10,856,529	3.18	9.6–67.3	5.01–11.33	4.69	-8.99	-4.32
	**Chr_14**	**26.7**	**Chr_14:5,297,579**	**5.09**	**13.9–40.0**	**2.04–7.45**	**5.90**	**-12.16**	**-3.38**
	Chr_19	83.6	Chr_19:10,727,069	3.46	71.7–114.2	8.76–13.78	5.67	-30.87	20.92
GSI	Chr_1	164.0	Chr_1:22,807,065	3.55	0.0–167.4	0.17–22.90	3.51	-0.68	0.51
	Chr_8	48.6	Chr_8:10,856,529	3.37	9.6–67.3	5.01–11.33	5.25	-0.69	-0.45
	**Chr_14**	**26.7**	**Chr_14:5,297,579**	**4.33**	**8.7–48.9**	**1.30–7.97**	**4.75**	**-0.82**	**-0.21**
	Chr_19	83.6	Chr_19:10,727,069	3.40	60.3–114.2	6.47–13.78	5.94	-2.48	1.83

Significant QTLs (*p* < 0.05) are highlighted with bold font

^a^ID of the SNP nearest the log of odds (LOD) peak position

^b^LOD score at peak position

^c^95% bayesian credible interval (CI)

^d^Phenotypic variation explained (PVE)

^e^Estimated additive effect from the grandmother alleles

^f^Estimated dominance effect from the grandmother alleles

## Candidate Gene Search

Maturation and/or body size related genes residing in the 95% CI of the significant QTL on Chr_14 (13.9 cM to 40.0 cM, corresponding to 2.0 to 7.5 Mbp) and Chr_21 (0 cM to 13.7 cM, corresponding to 0.1 to 3.7 Mbp) were picked up from the Fugu genome assembly (FUGU5/fr3) (Table 6). All predicted protein-coding genes (Chr_14: 232 genes; Chr_21: 125 genes) are listed in Online resource 3. Among them, we listed the following genes as candidate genes underpinning the early onset of testis development and large body size seen in the precocious family line: fibroblast growth factor 18α (*fgf18a*) and growth differentiation factor 9 (*gdf9*) on Chr_14 and bone morphogenetic factor 3 (*bmp3*) and fibroblast growth factor 5 (*fgf5*) on Chr_21.

**Table 6:** Maturation- and growth-related genes located in the 95% credible interval of significant QTLs

Chromosome	Gene symbol	Gene name
Chr_14	*fgf18a*	Fibroblast growth factor 18a
	*egr1*	Early growth response 1
	*gdf9*	Growth differentiation factor 9
	*nsdhl*	NAD(P) dependent steroid dehydrogenase-like
	zgc:194246	(Predicted to be involved in spermatogenesis)
Chr_21	*aplnra*	Apelin receptor a
	*rflna*	Refilin A
	*hmgcra*	3-Hydroxy-3-methylglutaryl-CoA reductase a
	*isl1*	ISL LIM homeobox 1
	*kazald3*	Kazal-type serine peptidase inhibitor domain 3
	*bmp3*	Bone morphogenetic protein 3
	*fgf5*	Fibroblast growth factor 5
	*bmp2k*	BMP2 inducible kinase

## Discussion

In this study, we investigated the utility of GRAS-Di for genetic studies, i.e., family structure analysis, genetic linkage map construction, and QTL analysis, using a cultured population of the tiger pufferfish. Approximately 22 K putative SNPs were obtained, which could resolve the fine-scale population structure and accurately assign parent-offspring pairs even for the very closely related families. A genetic linkage map was constructed using 1423 effective SNPs. The total length of the genetic map constructed from these effective SNPs was approximately 1950 cM. This is equivalent to the map size previously constructed with 1,220 microsatellite markers (2,200 cM) (Kai et al. 2011). Since a tissue sample from GM was not provided by the commercial hatchery, we could not fully utilize the polymorphisms between GF and GM, thus limiting the number of effective SNPs. The effective SNPs were also available for QTL mapping. As expected, individuals that inherited GF alleles at QTLs had larger testes. These results indicate that GRAS-Di is applicable for genetic analysis of aquaculture populations. Although SNP arrays are a much simpler and more reproducible genotyping platform than GBS (Robledo et al. 2018a), they require a large initial investment to design custom arrays from scratch and are currently available only for a limited number of aquatic species, such as Atlantic salmon (*Salmo salar*) (Houston et al. 2014; Yáñez et al. 2016; Bangera et al. 2017), common carp (*Cyprinus carpio*) (Xu et al. 2014), giant tiger shrimp (*Penaeus monodon*) (Baranski et al. 2014), catfish (*Ictalurus punctatus* and *I. furcatus*) (Liu et al. 2014; Zeng et al. 2017), Pacific oyster (*Crassostrea gigas*) (Qi et al. 2017; Gutierrez et al. 2018), European seabass (*Dicentrarchus labrax*) (Faggion et al. 2019), and Nile tilapia (*Oreochromis niloticus*) (Joshi et al. 2018; Peñaloza et al. 2020). Therefore, while GBS technologies are still the primary choice for population-scale genotyping in aquaculture species (Robledo et al. 2018a; Bresadola et al. 2020; Houston et al. 2020), GRAS-Di can be a viable and less expensive alternative choice for a genotyping platform.

Upon searching for maturation/growth related genes among the 232 and 125 predicted protein-coding genes within the 95% CI region of the two significant QTLs on Chr_14 and Chr_21, respectively, we found *gdf9* (for both maturation and growth) and *fgf18a*, *bmp3*, and *fgf5* (for growth). Notably, GDF9, a member of the transforming growth factor beta (TGFβ) superfamily, was first found as an oocyte-specific growth factor in vertebrates (McPherron and Lee 1993; McGrath et al. 1995) and is known as one of the control factors of steroid hormones: down-regulating estradiol and progesterone while up-regulating androgen (Solovyeva et al. 2000; Vitt et al. 2000; Spicer et al. 2006; Orisaka et al. 2009). In addition, GDF9 also acts on germ or somatic cells and controls spermatogenesis in vivo (He et al. 2012; Guo et al. 2013; Yan et al. 2020). In a previous study, we found that the precocious trait of the male tiger pufferfish was characterized by high levels of plasma estradiol-17β (E2) prior to the initiation of testicular development (Yoshikawa et al. 2020). Thus, polymorphisms in *gdf9* (and/or its regulatory region) may have enhanced E2 synthesis, leading to the precocious maturation of the family line. The TGFβ superfamily (BMP3) and fibroblast growth factor family (FGF5 and FGF18a) are known growth-related genes that play important roles in a variety of biological processes including cell proliferation, embryonic development, and bone/cartilage formation (Clase et al. 2000; Daluiski et al. 2001; Haque et al. 2007). These genes may also be responsible for the large body size of the precocious family line.

A significant QTL shared between TW and BW was detected on Chr_14. This is consistent with the previous finding that these traits showed a positive phenotypic correlation in the precocious family line that GF belonged to (Yoshikawa et al. 2020). These results suggest that the two traits have at least a partially common genetic architecture. Thus, it may be possible that both these genetic traits could be improved simultaneously. However, the genetic effect (i.e., PVE) of the significant QTL and other suggestive QTLs were small, and these values could even have been overestimated with the small number of samples, due to Beavis effect (Beavis 1994, 1998; Xu 2003). Therefore, the precociousness and the large body size seen in the elite family line seem to be polygenic and it would be difficult to directly integrate these QTLs into the selective breeding programs of the tiger pufferfish via marker-assisted selection. Instead, genomic selection (GS) is a better choice. GS is a selective breeding technology where genomic breeding values of selection candidates are predicted using linear regression linking genotypes and phenotypes of related individuals (Meuwissen et al. 2001). It has been integrated in some aquaculture species, including Atlantic salmon (Ødegård et al. 2014; Tsai et al. 2015; Robledo et al. 2018b), Yesso scallop (*Patinopecten yessoensis*) (Dou et al. 2016), Pacific oyster (Gutierrez et al. 2018), and coho salmon (*Oncorhynchus kisutch*) (Barría et al. 2018). Although hundreds of thousands of SNPs are required to conduct GS for livestock animals (Fan et al. 2010), it appears that a few thousand of SNPs are sufficient to gain high prediction accuracy in aquaculture species where cultured populations often consist of closely related individuals (Zenger et al. 2019; Kriaridou et al. 2020). At the population level, we were able to detect 22 K SNP loci using GRAS-Di in our rather small population, and therefore, we expect that GRAS-Di can be applied for GS of aquaculture species.

## Conclusion

Our results demonstrate that GRAS-Di is a practical genotyping tool for genetic studies of the cultured tiger pufferfish, and therefore, presumably useful for other aquaculture species. We were able to detect accurate population structure and construct a fine-scale linkage map construction. Furthermore, we could dissect small effect QTLs for body size and maturation-related traits of the population, suggesting that the precociousness is polygenic, and GS is the better choice for a breeding strategy for this trait rather than marker-assisted selection.

## Supplementary Information

Below is the link to the electronic supplementary material.

Supplementary file1 (XLSX 964 KB)

Supplementary file2 (XLSX 11 KB)

Supplementary file3 (XLSX 27 KB)

## Data Availability

All raw sequencing data have been deposited in the DDBJ Sequence Read Archive database (Accession No. DDBJ: DRA010711).

## Abbreviations

BW: Body weight
CI: Credible interval
E2: Estradiol-17β
F_1_: First generation
F_2_: Second generation
FA1: Father 1
FA2: Father 2
GBS: Genotyping-by-sequencing
GF: Grandfather
GM: Grandmother
GRAS-Di: Genotyping by random amplicon sequencing, direct
GS: Genomic selection
GSI: Gonadosomatic index
LOD: Log of odds
MO1: Mother 1
MO2: Mother 2
NPIF: Nagasaki Prefectural Institute of Fisheries
PIT: Passive integrated transponder
PVE: Phenotypic variation explained
QTL: Quantitative trait locus
SL: Standard length
SNP: Single-nucleotide polymorphism
SSR: Simple-sequence repeats
TW: Testes weight
